# Decoding the Raf-Mek-Erk-Rsk pathway in prostate cancer: from molecular mechanisms to clinical opportunities

**DOI:** 10.1038/s41416-026-03441-x

**Published:** 2026-05-13

**Authors:** Nick R. Waldron, Diogo Silva, Daniel Westaby, Juan Jiménez-Vacas, Joe Taylor, Adam Sharp

**Affiliations:** 1 The Institute of Cancer Research, London, UK; 2 The Royal Marsden NHS Foundation Trust, London, UK; 3 Translational and Clinical Research Institute, Centre for Cancer, Newcastle University, Newcastle upon Tyne, UK

**Keywords:** Prostate cancer, Molecular medicine

## Abstract

Advanced prostate cancer remains a major healthcare problem and cause of death in the United Kingdom. In contrast to many other cancer types, with the exception of poly (ADP-ribose) polymerase inhibition in DNA repair defective cancers, clinically actionable molecular subtypes are lacking. There are a number of studies that suggest the RAF-MEK-ERK-RSK cascade, a major oncogenic pathway, is activated in prostate cancer and this increases as the disease progresses. Mechanisms of activation are not dominated by pathogenic mutations in pathway proteins and include paracrine and autocrine mechanisms. There is strong evidence linking pathway activation with enhancing key proliferative signalling programmes and promoting cell survival in prostate cancer. Whilst inhibitors of the RAF-MEK-ERK-RSK pathway have demonstrated clinical utility in other cancers this has not been realised in prostate cancer. The reasons for this include a lack of predictive biomarkers for, and the unique landscape of pathway activation. Future studies need to identify robust predictive biomarkers, and improve the understanding of the fundamental biology of RAF-MEK-ERK-RSK activated prostate cancers if we are to successfully target this important sub-group.

## Introduction

Prostate cancer is the most common malignancy in those assigned male sex at birth in the United Kingdom, and metastatic disease accounts for around 13,000 deaths per year [1]. The initial phases of advanced disease are usually driven by the androgen receptor (AR) and are responsive to therapies targeting the AR signalling pathway (termed castration sensitive prostate cancer, CSPC). Almost inevitably, tumours will evolve mechanisms to resume proliferation in a low androgen environment, termed castration resistant prostate cancer (CRPC) [2]. This is driven most often through persistent AR signalling, but can also be through AR independent mechanisms. In many other tumour types, distinct clinically actionable molecular subsets have been identified that allow for more precise treatment and improved outcomes. However, in CRPC, the poly (ADP-ribose) polymerase inhibitor olaparib is the only biomarker-directed therapy currently licensed and funded for use in the National Health Service [3]. Thus, there is an urgent unmet need to identify clinically relevant molecular subsets of CRPC that respond to targeted therapies, in novel biomarker-directed approaches. One such attractive subset could be those patients with activation of the RAF-MEK-ERK-RSK pathway.

## The RAF-MEK-ERK-RSK pathway

Mitogen-activated protein kinase pathways (MAPK), of which there are four classical family cascades (ERK, c-Jun N-terminal kinase, p38 mitogen-activated protein kinase, extracellular signal-regulated kinase 5) in mammals, form a critical module in signal transduction pathways that influence diverse cellular processes, including proliferation, metabolism, survival, and motility [4].

Of the four MAPK cascades, the RAF-MEK-ERK-RSK pathway is the most studied with respect to dysregulation in cancer, and will be the focus of this review article (referred to as MAPK from here on in) [5]. It can be activated either through cell surface receptor-ligand interactions (ligand-dependent, with receptor tyrosine kinases, including the epidermal growth factor receptor family and the platelet-derived growth factor receptors [6, 7], and G protein-coupled receptors being the key effectors), physical stressors or constitutive activation of component proteins through activating mutations (ligand-independent) [8].

**Fig. 1:**
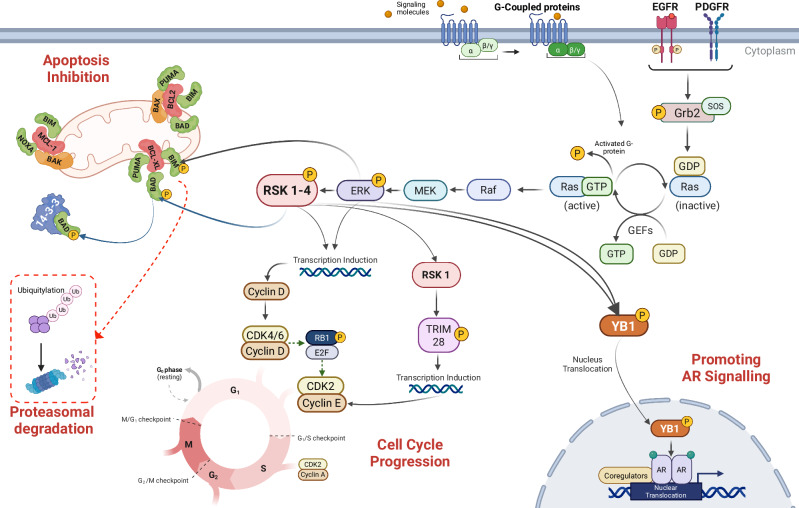
The MAPK cascade and its role in sustaining proliferative signalling and promoting cell survival in prostate cancer cells. *Created in BioRender. Silva, D. (2026)* https://BioRender.com/jqkn0d4. The MAPK pathway cascade is initiated by ligand dependent and independent means. In ligand dependent activation as illustrated, signalling via cell surface receptors such as EGFR or G-coupled protein receptors initiates the phosphorylation cascade. Activation of the MAPK pathway is implicated in prostate cancer development, proliferation and treatment resistance. MAPK signalling interacts with the intrinsic apoptosis proteins to promote a pro-survival phenotype. This is through both increased expression of anti-apoptotic proteins and down-regulation (through post-translational modifications) of pro-apoptotic members. MAPK activation drives cell proliferation through promoting cell cycle progression, especially at G1/S, in RB dependent (transcriptional induction of Cyclin D) and RB independent (RSK phosphorylation of TRIM28) mechanisms. Finally, evidence suggests MAPK driven phosphorylation of YB1 may promote the AR transcriptional programme.

In ligand-dependent activation, the initial step in signal transduction is the formation of the growth factor receptor binding protein 2 and son of sevenless complex [9, 10]. This complex facilitates guanine nucleotide exchange on rat sarcoma (RAS), cycling RAS from its inactive GDP bound state to its active GTP one [11]. RAS is then able to activate the three isoforms of RAF (ARAF, BRAF and CRAF) and induce dimerization to fully activate its kinase domain. Activated RAF phosphorylates MEK, which in turn phosphorylates and activates ERK. Once activated, ERK modulates the function of many cellular processes through phosphorylation of various substrates, either directly or through activation of other downstream protein kinases. One key set of effector proteins are the ribosomal s6 kinase protein family (RSKs), of which there are four isoforms, RSK1-4 [12]. Phosphorylation of RSK by ERK is a crucial step in its activation. Negative regulation of the pathway occurs at a number of levels, including direct upstream negative feedback loops, and transcriptional induction of MAPK pathway inhibitors with the dual-specificity MAPK phosphatases (DUSPs) being the most studied. DUSP proteins dephosphorylate and thus deactivate ERK, as well as sequester it in an inactive form to reduce MAPK signalling [13, 14] [Fig. 1].

## Pathway overactivation: pan-cancer

Mutations within the RAS genes (of which there are three isoforms, KRAS, NRAS, and HRAS) represent the most common mechanism of pathway activation in cancer, with KRAS mutations highly prevalent in adenocarcinomas of the pancreas (~95%), colon (~40%), and lung (~30%) [15, 16]. These mutations typically occur at codons 12, 13, or 61, leading to constitutively active GTP-bound RAS, which bypasses normal regulatory controls and persistently activates downstream MAPK signalling [17].

Mutations in BRAF also contribute significantly to oncogenic activation. The most common pan-cancer mutation is BRAF V600E, which is particularly prevalent in melanoma (40-60%), papillary thyroid carcinoma (~45%), and colorectal cancer (~10%). It leads to constitutive activation independently of upstream RAS input, driving sustained proliferation and tumour survival mechanisms [18, 19].

Dysregulation of phosphatases and regulatory proteins also facilitates pathway hyperactivation. Loss-of-function mutations or epigenetic silencing of DUSPs allow persistent ERK activation, promoting oncogenesis [20]. Moreover, the functional inactivation of neurofibromin 1, a RAS-GTPase activating protein, contributes to sustained pathway signalling, particularly notable in melanoma and neurofibromatosis type 1-associated tumours [21].

## Pathway overactivation: prostate cancer

MAPK activation has been implicated in both the development and progression of prostate cancer and associates with poor clinical outcomes. In 1986, Viola and colleagues performed RAS immunohistochemistry (IHC) in normal, benign hyperplastic, and cancerous prostate tissue. Expression was absent in normal and benign lesions, with increasing likelihood of positivity with higher cancer grade [22]. A further study that looked at the three different RAS isoforms in paired castration sensitive and castration resistant biopsies (all of prostatic origin), found that increasing NRAS expression at CRPC, but not HRAS or KRAS, was predictive of shorter disease-specific survival [23]. In contrast, a third study found no association between RAS staining and tumour grade or clinical stage. A potential explanation for the conflicting results may be due to limited antibody specificity of earlier RAS antibodies [24, 25]. Few studies to date have looked specifically at RAS protein expression in metastatic lesions, although one study found greater staining intensity in bone versus lymph node metastases [26].

IHC studies have also demonstrated increased ERK activation (as determined by ERK phosphorylation) in malignant prostate tissue as compared with normal prostate epithelium. Increased phosphorylated ERK staining correlates with higher Gleason score and more advanced clinical presentation at diagnosis [27, 28]. Nickols and colleagues performed Virtual Inference of Protein-activity by Enriched Regulon (VIPER) analysis on 101 CRPC biopsies, and compared to primary prostate cancer samples, found 7 kinases with inferred higher activation, including ERK [29]. A limitation of these datasets is reliance on prostate tissue rather than metastatic site biopsies, as all CRPC biopsies came from transurethral resection of the prostate. In the largest characterisation of metastatic lesions, Li and colleagues did show higher ERK phosphorylation staining in a set of CRPC biopsy tissue microarrays as compared to a localised disease tissue microarray [30]. Downstream of ERK, expression of RSK1 and RSK2 are around 3.5 times higher in prostate cancer specimens as compared to normal prostatic epithelium. Interestingly, positive phosphorylated RSK staining was demonstrated to be more likely in bone metastases than metastases from lymph nodes (40% versus 5%) [31, 32].

Unlike in melanoma, colorectal and lung cancer, activating mutations in key proteins of the MAPK cascade in advanced prostate cancer are relatively rare. BRAF is the most commonly mutated member of the pathway, occurring at a frequency of around 2-3%. In contrast to other tumour types, BRAF K601E and G469A are more common than V600 mutations, and structural variants make up around half of the genomic aberrations [33, 34]. RAS, MEK, ERK and RSK mutations are relatively rare in CRPC, however, amplification in pathway members is more common. RSK2 is amplified in around 10% CRPC, while around 5% of cases have KRAS and BRAF amplification. In terms of negative regulators, DUSP4 is the most commonly deleted in around 10%. Rates of RAF, RAS and DUSP4 aberrations appear to be similar in primary prostate cancer and castration resistant disease, suggesting they may be early events. In contrast, RSK2 amplification is around 10 times more common in CRPC [35, 36] [Table 1].

**Table 1:** Genomic aberrations in individual proteins of the MAPK cascade are rare in prostate cancer.

Gene	Aberration	Frequency
HRAS	Amplifications	2%
	Point mutations	<1%
NRAS	Amplifications and point mutations	1-2%
KRAS	Amplifications	2%
	Point mutations	2%
BRAF	Amplifications	3-5%
	Point mutations	3%
	Fusions	3%
MEK	Amplifications and point mutations	1-2%
ERK	Amplifications and point mutations	1-2%
DUSP4	Homozygous loss	10%
RSK1	Any aberration	<1%
RSK2	Amplifications	10%
RSK3	Amplifications	2%
	Homozygous loss	2%
RSK4	Amplifications	2%
	Homozygous loss	2%

For each gene, the aberration type and frequency (percentage of cases affected) is displayed.

As advanced prostate cancer is a disease more commonly characterised by copy number alterations than individual point mutations, others have tried more integrated genomic analysis to account for this. In a large dataset of 181 primary prostate cancer samples, 37 metastatic samples, and 12 prostate cancer cell and xenograft models, Taylor and colleagues interrogated both copy number alterations and transcriptomic profiling. Combining these data sets, the MAPK pathway was much more frequently altered (43% of primary tumours and 90% of metastatic tumours) [37].

Alternatively, ERK signalling may be activated in a paracrine fashion through various mechanisms. It has recently been demonstrated that infiltrating myeloid-derived suppressor cells (MDSCs) in the prostate tumour microenvironment enhance androgen independent tumour growth by secreting coagulation factor X, which activates ERK signalling via protease-activated receptor-2 (PAR2) [38]. Chemokines and chemokine receptors play a key role in trafficking MDSCs into the tumour microenvironment. The atypical chemokine receptor 3 has been shown to activate MAPK signalling to mediate resistance to the AR pathway inhibitor enzalutamide, although the mechanism of activation here was suggested to be stabilisation of the MAPK scaffold protein beta-arrestin rather than recruitment of pro-inflammatory tumourigenic immune cells [30].

There is also evidence of autocrine feedback loops in prostate cancer cells activating MAPK signalling. Using single cell RNA sequencing, Steiner and colleagues identified lymphocyte antigen 6 family member D as a cell surface marker for a subset of intrinsically castration resistant prostate cells. Tumours grown from these cells were found to activate ERK through a self-amplifying secretion of amphiregulin, signalling through the epidermal growth factor receptor, with growth in 3D models reduced by MAPK inhibition [39].

It has also been demonstrated that in models of double negative prostate cancer – where there is loss of AR expression but no evidence of histological differentiation to a neuroendocrine or small cell state – there is elevated MAPK signalling mediated through fibroblast growth factors. Furthermore, MEK inhibition reduced the proliferation of both in vitro and in vivo double negative models [40]. Similarly, analysis of a multi-omics dataset suggests that the activation of the MAPK pathway promotes lineage plasticity in prostate cancer, suggesting a role for MAPK activation in bypassing AR signalling [41].

Epigenetic modifications in advanced prostate cancer may also contribute to MAPK activation. The transcription factor reduced expression-1 has been shown to increase phosphorylated ERK levels in PC3 and LNCaP prostate cancer cell lines. Mechanistically, this was through DNA methyltransferase 3 beta facilitated promoter methylation of the inhibitory Ras association domain family member 1 A [42].

In terms of clinical outcomes, higher MAPK activation at CRPC compared to primary tissue (nuclear expression of phosphorylated ERK, CRAF expression) is reported to be predictive of shorter survival time following relapse from early stage disease and reduced disease specific survival [43, 44]. However, at present there is little published data beyond associations between IHC, genomic and transcriptomic data and clinical variables with which to draw firm conclusions about how MAPK activation influences key clinical outcomes. One ongoing study is attempting to evaluate a MAPK/ERK gene signature and phosphorylated ERK IHC in predicting treatment responses, although to our knowledge no results are as yet available [45].

Overall, there is a wealth of data to support genomic, transcriptomic and protein level aberrations of key MAPK proteins in prostate cancer. The extent of aberrancy usually increases as disease progresses from premalignant, to primary cancer, to CRPC. There is also intriguing data linking activated MAPK pathway with lineage plasticity and differentiation to AR independent phenotypes. The main limitation is the lack of studies assessing the functional consequences of these alterations; most available data are correlative, and no prospective studies have evaluated markers of MAPK activation as a predictive or prognostic biomarker.

## Oncogenic consequences of pathway activation

Aberrant activation of the pathway as described above is frequently observed in diverse cancers and significantly contributes to disease progression, therapeutic resistance, and adverse patient outcomes, through multiple oncogenic consequences.

### Sustaining proliferative signalling, growth and senescence

There is strong evidence linking activation of the MAPK pathway with progression through the cell cycle, particularly at the G1/S checkpoint. Multiple studies have demonstrated MAPK activation promotes G1/S progression. Conversely ERK knockout or pharmacological inhibition leads to almost complete abrogation of entry to S phase [46–50]. Mechanistically, pathway activation leads to phosphorylation of three transcription factors from the ETS family (Ets-like proteins 1, 3 and 4). This leads to transcriptional induction of cyclin D1 which complexes with cyclin-dependent kinases 4 and 6 [51, 52]. These complexes phosphorylate and deactivate retinoblastoma protein (RB), releasing the E2F transcription factors to activate cyclin E, ultimately leading to S phase entry. The activity of the CDKs is opposed by cyclin-dependent kinase inhibitors (CDKIs), including p21 and p27kip1 [Fig. 1]. Both ERK and RSK are implicated in repressing the activity of the CDKIs through multiple mechanisms [53, 54]. Pharmacological inhibition of the pathway (for example with trametinib, a MEK inhibitor) leads to induction of p27kip1, most markedly in cell lines with constitutive pathway activation [50].

Whilst MAPK activation can promote cellular proliferation and progression through the cell cycle, it is usually insufficient to drive uncontrolled proliferation. For example, prolonged expression of RAS in primary human or murine fibroblasts leads to sustained cell cycle arrest and a senescent state through induction of TP53 and cyclin-dependent kinase inhibitor 2 A; BRAF V600E mutation is found in up to 80% of melanocytic naevi but this alone is not sufficient to induce malignant transformation [55, 56]. Generally, it has been demonstrated that overcoming this senescent state requires loss of a co-operative tumour suppressor such as TP53, cyclin-dependent kinase inhibitor 2 A or PTEN [57, 58]. Indeed, in murine models, KRAS G12D mutation alone is insufficient to induce prostate cancer, whereas when combination with PTEN loss leads to an aggressive oncogenic phenotype [59].

In other words, MAPK-driven proliferation is often restrained by cellular senescence, and additional genomic alterations or cellular reprogramming are typically required to reactivate the proliferative program.

In the majority of advanced prostate cancers, the AR signalling pathway remains the key driver of growth and proliferation [60]. There is evidence linking activation of the MAPK pathway with sustaining AR signalling. Human epidermal growth factor receptor 2 overexpression in the hormone sensitive prostate cancer cell line LNCaP has been shown to increase expression of the AR downstream marker prostate specific antigen (PSA). This increase in PSA was abrogated with the addition of a MEK inhibitor, suggesting in this context at least, the existence of a possible human epidermal growth factor receptor-MAPK-AR axis [61].

Y-box-binding protein-1 (YB-1) is another possible downstream effector linking MAPK activation and AR signalling. When phosphorylated by either activated ERK and RSKs at serine 102, nuclear translocation is observed. YB-1 has been suggested to increase AR expression transcriptionally by binding to the AR promoter [62]. RSK2 has been shown to enhance PSA expression through increasing AR-mediated transcription in LNCaP cells that have been serum starved, whilst a RSK inhibitor reduced their growth [31] [Fig. 1].

There is perhaps a greater weight of evidence supporting a role for MAPK activation in promoting the castration resistant phase of growth (which in many cases is driven by persistence of AR signalling). Overexpression of YB-1 has been shown to induce castration resistance in LNCaP cells [63, 64]. Li and colleagues showed an increase in phosphorylated ERK in enzalutamide-resistant prostate cancer cell lines versus parental controls, and corresponding synergy when combining trametinib with enzalutamide in these models [30]. However, the doses of trametinib used (1 µM and above) are much higher than the typical dose needed to completely suppress phosphorylated ERK (around 10 nM in prostate cancer cell lines in our hands and others) [65]. Paracrine ERK activation from the tumour microenvironment has also been linked with promoting castration resistance. Wang and colleagues isolated a subset of CRPC specific cancer associated myofibroblasts using single cell RNA-sequencing and demonstrated that their secretion of osteopontin led to paracrine activation of ERK. Combined inhibition of ERK and AR signalling prevented this osteopontin-mediated, ERK-dependent, development of castration resistance in murine tumours [66].

The MAPK pathway likely also plays a role in prostate cancer cell proliferation through mechanisms other than AR signalling. SL0101, a RSK inhibitor, reduced proliferation in the AR-negative PC3 cell line, whilst others have implicated RSK isoforms in driving G2/M progression in these cells [31, 67]. Treatment of the BRAF L597R mutant, enzalutamide-resistant CRPC 22Rv1 cell line with the RSK inhibitor PMD-026, in combination with enzalutamide, induced G2/M arrest and reduced both in vitro and in vivo growth compared to either agent alone, but did not affect full-length AR at the protein or RNA level [68]. Recently, Kim and colleagues demonstrated that TRIM28, when phosphorylated by RSK1, can promote transcriptional activation of E2F and drive cell cycle progression in prostate cancer models independently of RB status, including the AR-negative DU145 cell line. Accordingly, treatment with the RSK inhibitor BI-D1870 abolished this RSK-TRIM28-E2F loop and reduced growth in multiple prostate cancer cell lines and in murine models bearing LuCaP145.1 tumours (AR negative, neuroendocrine prostate cancer model) [69].

### Cell survival

Apoptosis is a form of regulated cell death and occurs through two pathways (extrinsic and intrinsic). Several studies have linked activated ERK signalling with inhibition of the intrinsic pathway. The intrinsic pathway is controlled by the BCL-2 family of proteins, of which there are three key subgroups: pro-apoptotic pore-forming proteins (BAX, BAK, and BOK); pro-apoptotic BCL-2 Homology domain 3 (BH3)-only proteins (BAD, BID, BIM, PUMA, and NOXA amongst others); and anti-apoptotic proteins (of which MCL-1, BCL-XL, BCL-2 are the most studied). The balance and binding between these proteins determines whether a cell will undergo apoptosis; ultimately if the pore forming effector proteins are inhibited by the anti-apoptotic members, cell survival will be favoured, and vice versa [70].

**Fig. 2:**
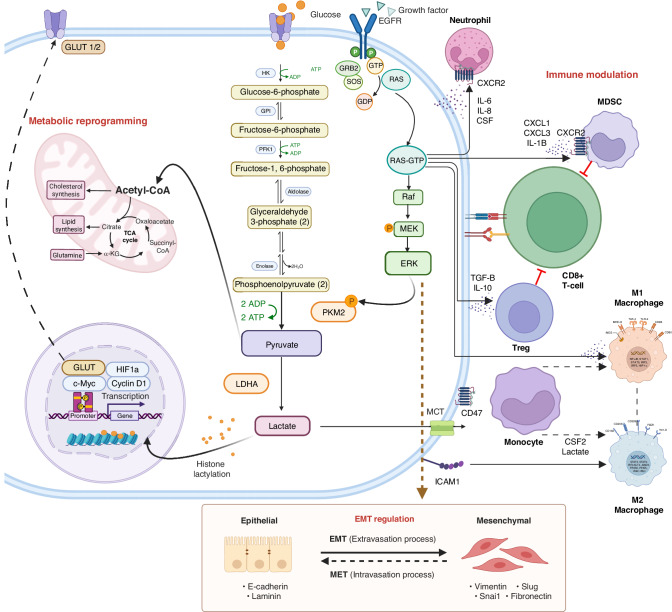
Impact of MAPK signalling on metabolic reprogramming, immune modulation, and epithelial-mesenchymal plasticity in prostate cancer. *Created in BioRender. Silva, D. (2026)* https://BioRender.com/p5w1x5c. MAPK pathway activation downstream of EGFR promotes oncogenic signalling through the MAPK cascade, coordinating multiple tumor-promoting processes: (1) Metabolic reprogramming: ERK activation enhances glycolytic flux via increased GLUT1/2 expression and glucose uptake. Glycolysis proceeds through sequential phosphorylation steps (GPI, PFK1, aldolase, enolase) generating pyruvate, which is phosphorylated by PKM2 and converted to lactate by LDHA. Lactate promotes histone lactylation, activating transcription factors (HIF1α, c-Myc, Cyclin D1) that amplify GLUT expression and glycolytic enzyme production. Acetyl-CoA promotes lipid and cholesterol synthesis pathways via the TCA cycle, supporting rapid tumour cell proliferation. (2) Immune modulation: MAPK signalling regulates the tumour microenvironment through multiple mechanisms: ERK promotes secretion of immunosuppressive cytokines (IL-6, IL-8, CSF, IL-1β, TGF-β, IL-10) and chemokines (CXCL1, CXCL3) that recruit myeloid-derived suppressor cells, regulatory T cells, and tumour-associated macrophages. Neutrophil recruitment occurs via CXCR2 signaling. Lactate export through MCT1 creates an immunosuppressive acidic microenvironment, promoting M2 macrophage polarisation and CD8+ T-cell exhaustion. Immune checkpoint molecules (CD47, ICAM1) and M2 macrophage markers (CSF2, lactate sensing) facilitate immune evasion. (3) Epithelial-mesenchymal transition regulation: The pathway modulates epithelial-mesal markers (vimentin, Snai1, Slug, fibronectin) that control invasion and metastasis. This integrated network highlights therapeutic opportunities for MAPK inhibitors in disrupting the metabolic-immune-metastatic axis in prostate cancer. CD47, cluster of differentiation 47; CD8+ , cluster of differentiation 8-positive; CSF, colony-stimulating factor; CSF2, colony-stimulating factor 2; CXCL1/3, C-X-C motif chemokine ligand 1/3; CXCR2, C-X-C chemokine receptor type 2; EGFR, epidermal growth factor receptor; EMT, epithelial-mesenchymal transition; GLUT1/2, glucose transporter 1/2; GPI, glucose-6-phosphate isomerase; GRB2, growth factor receptor-bound protein 2; HIF1α, hypoxia-inducible factor 1-alpha; HK, hexokinase; ICAM1, intercellular adhesion molecule 1; IL-1β, interleukin-1 beta; IL-6, interleukin-6; IL-8, interleukin-8; IL-10, interleukin-10; LDHA, lactate dehydrogenase A; M1/M2, macrophage phenotype 1/2; MCT1, monocarboxylate transporter; MDSC, myeloid-derived suppressor cell; MET, mesenchymal-epithelial transition; PFK1, phosphofructokinase 1; PKM2, pyruvate kinase M2; TCA, tricarboxylic acid cycle; TGF-β, transforming growth factor beta; Treg, regulatory T cell; α-KG, alpha-ketoglutarate.

Activated ERK has been shown to phosphorylate the pro-apoptotic protein BIM at serine 69, promoting both proteasomal degradation and dissociation from the anti-apoptotic proteins MCL-1 and BCL-XL, activating them and promoting cell survival [71, 72]. Indeed, it has been demonstrated that apoptosis induction in response to MEK inhibition in both KRAS mutant non-small cell lung cancer cells and BRAF V600E mutant melanoma cells is dependent on an increase in BIM levels [73, 74]. ERK also directly phosphorylates MCL-1 at both threonine 92 and 163, which conversely stabilises this anti-apoptotic protein [75]. The MAPK pathway is also implicated in the regulation of the two other key anti-apoptotic proteins, BCL-2 and BCL-XL. In the Ba/F3 pro-B lymphocyte cell line, MAPK phosphorylation of the GATA binding protein 1 led to induction of BCL-XL expression, whilst MEK inhibition in pancreatic and mucosal melanoma cancer cells down-regulated MCL-1, BCL-2 and BCL-XL [76–78]. ERK signalling also leads to activation of the transcription factor NFκB, which has been shown to up-regulate BCL-XL expression, promoting survival [79] [Fig. 2]. NOXA has also been suggested to be a key player in regulating apoptotic responses to targeted therapies, including MAPK inhibitors. In a study utilising multiple oncogene-activated cell lines, including those with BRAF mutations, Montero and colleagues demonstrated a common response of NOXA depletion to the relevant oncogene-targeted therapy, coupled with an increased sensitivity to MCL-1 inhibition [80]. Importantly, the effects were specific to when the targeted therapy matched the cell’s corresponding oncogene addiction. Downstream of ERK, the RSK proteins phosphorylate BAD at serine-112, which allows it to be sequestered by 14-3-3, preventing it from binding and deactivating the anti-apoptotic proteins BCL-XL and BCL-2 [81] [Fig. 1].

There has been very little study of the potential interaction between MAPK signalling and the intrinsic apoptosis pathway specifically in prostate cancer models. Zelivianski and colleagues used a combination of docetaxel and MAPK inhibitors in LNCaP and PC3 cells, with the combination modestly increasing apoptosis versus single agent treatment, putatively through inactivation of BCL-2 [82]. The effects were likely modest as neither of these models have been shown to be MAPK-overactivated. In DU145 prostate cancer cells, which harbour a UBE2L3-KRAS fusion [83], the isoprenoid Farnesol (a naturally occurring organic hydrocarbon found in food such as peaches, tomatoes and lemongrass) was shown to inhibit ERK activation, and trigger apoptosis probably through multiple mechanisms including upregulation of BAX and downregulation of BCL-2 [83, 84]. Finally, docetaxel-resistant cell lines derived from DU145 show increased resistance to apoptosis through further upregulation of ERK1/2, a phenotype partially abrogated by siRNA knockdown of c-MYC oncogene [85].

Whilst the evidence above certainly supports the notion that MAPK signalling has important pro-survival functions, oncogenic cellular evolution and context are key. Other signalling pathways, notably the PI3K/AKT pathway, similarly promote survival in cancer cells, often with mechanistic redundancy between the two pathways; as an example, BAD is phosphorylated at serine 136 by the AKT pathway and serine 112 by the ERK pathway, with similar functional effects [86, 87]. As the work by Montero and colleagues demonstrates, only by targeting the dominant signalling pathway are we likely able to significantly influence the balance and binding patterns of the BCL-2 family proteins to shift a cell’s fate towards apoptosis [80].

### Cancer metastasis and epithelial-to-mesenchymal transition

Metastasis is significantly influenced by the activation of the MAPK pathway through its role in epithelial-to-mesenchymal transition (EMT). EMT is characterised by loss of epithelial characteristics and acquisition of mesenchymal traits, such as increased migratory and invasive capabilities. MAPK activation orchestrates the nuclear accumulation of EMT-promoting transcription factors, including Snail, Twist, and ZEB family proteins, while concomitantly suppressing epithelial markers (such as E-cadherin) and upregulating mesenchymal markers (N-cadherin, vimentin, fibronectin) [88, 89]. This phenotypic switch not only enhances the invasive potential of cancer cells but also contributes to their resistance to chemotherapy and immune surveillance [90].

Evidence from genetically engineered murine models of prostate cancer demonstrates that co-activation of RAS, particularly in the context of PTEN deficiency, robustly induces EMT, enhances stem/progenitor populations, and accelerates metastatic dissemination [59]. In vitro and ex vivo analyses corroborate that ERK pathway activation not only drives phenotypic switching but also upregulates matrix metalloproteinases, thereby facilitating tissue invasion and cell motility [91]. Pharmacological targeting of MAPK, or genetic depletion of key nodes such as ERK2, abrogates EMT marker expression and suppresses metastatic competency in preclinical models [59, 91]. In clinical samples (predominantly early stage disease represented), patients with high ERK activation (phospho-ERK IHC) had higher levels of SNAIL, an expression pattern that was associated with worse progression-free survival [92] [Fig. 2].

### Cancer metabolic reprogramming

Metabolic reprogramming is intimately linked to the MAPK pathway. Tumour cells exhibiting hyperactive ERK signalling demonstrate increased glycolytic flux, lactate production, and enhanced glucose uptake (Warburg effect). Specifically, ERK directly modulates glycolytic enzymes such as lactate dehydrogenase, hexokinase II and pyruvate kinase M2, promoting their enzymatic activity and nuclear translocation [93, 94]. Additionally, ERK pathway activation transcriptionally upregulates glucose transporters (GLUT1 and GLUT3) and enhances the expression of rate-limiting glycolytic enzymes through c-MYC oncogene and Hypoxia inducible factor-1α-dependent mechanisms [95, 96]. Furthermore, ERK pathway activation is involved in regulating mitochondrial metabolism, enhancing glutaminolysis through direct phosphorylation of glutamine metabolic enzymes, and promoting lipid biosynthesis pathways necessary to sustain rapid tumour growth and survival under nutrient-deprived conditions. Recent work demonstrates that lactate functions not merely as a metabolic byproduct but as an epigenetic regulator that sustains a metabolic-epigenetic axis in prostate cancer cells. This lactate-mediated pathway enhances lipid droplet formation, supports mitochondrial respiration through anaplerotic reactions, and promotes histone acetylation patterns that favour aggressive cancer phenotypes [97, 98].

In prostate cancer, metabolic adaptation is another consequence of MAPK pathway activation. In PTEN/p53-deficient murine models, ERK pathway activation correlates with enhanced lactate production and glycolytic reprogramming. They exhibited markedly increased lactate-generating signalling pathways that promoted tumour growth and immune evasion [99]. Dual PI3K and MEK inhibition in docetaxel-resistant CRPC models normalised glycolysis, reduced tumour growth, and induced apoptosis, suggesting that MAPK-driven metabolic rewiring supports chemoresistance and survival [100] [Fig. 2].

### Immune and inflammatory response modulation

The MAPK pathway also influences immune and inflammatory responses within the tumour microenvironment. Activation of the MAPK cascade stimulates the production and secretion of pro-inflammatory cytokines and chemokines, including interleukin-6 and interleukin-8. These factors promote chronic inflammation, angiogenesis, and immune suppression within the tumour milieu [101, 102]. Specifically, ERK signalling contributes to the polarisation of tumour-associated macrophages towards an immunosuppressive phenotype (M2-like macrophages), facilitating tumour immune evasion and creating a conducive environment for tumour growth and metastasis. Macrophage-derived cytokines including c-c motif chemokine ligand 3, interleukin-1 receptor antagonist, osteopontin, macrophage colony stimulation factor 1 and glial cell line-derived neurotrophic factor enhance cell proliferation through activation of ERK and AKT pathways, establishing a feed-forward loop that amplifies immunosuppressive signals [103]. The therapeutic potential of targeting MAPK-driven immune evasion is being studied in clinical trials, most commonly combined with immune checkpoint blockade. However, the phase III COMBI-I trial (dabrafenib plus trametinib with either placebo or the PD1 inhibitor spartalizumab) did not meet its primary endpoint [104].

Typically, a ‘cold’ tumour, prostate cancer exhibits immunosuppressive modulation via MAPK signalling. ERK-driven production of interleukin-6 and chemokine ligands contributes to the recruitment of myeloid-derived suppressor cells and regulatory T lymphocytes to the tumour microenvironment [105] [Fig. 2].

## Clinical utility of MAPK inhibition

Clinical efforts targeting the MAPK pathway have significantly evolved, leading to several National Institute approved therapies and ongoing investigations across cancer types. First-generation RAF inhibitors, specifically targeting V600E mutations (vemurafenib, dabrafenib, encorafenib) have notably transformed clinical management in melanoma, non-small cell lung cancer and colorectal cancer. These have all been approved following successful phase III clinical trials, either as monotherapy or in combination (e.g. with MEK inhibitors) [106].

Despite these advancements, resistance to RAF inhibitors has emerged as a significant clinical challenge. The most widely used classification system to conceptualise the oncogenic BRAF mutation landscape groups mutations based on biochemical and signalling properties has provided a helpful framework for next-generation drug discovery [107]. Class I mutations occur at the amino acid V600, which sits in the activation loop of the kinase domain. These mutations mimic activating phosphorylation of key residues in the activation loop, and allow BRAF to signal independently of upstream RAS [108–113]. Currently approved BRAF inhibitors bind to the exposed active site. Traditionally, this was thought to allow BRAF to signal as a monomer. Impairing dimerization using the R509H mutant did not affect the ability of V600 mutations to activate downstream signalling, in contrast to non V600 mutations [108]. Additionally, clinically effective first generation BRAF inhibitors only target monomeric BRAF (see below), and expression of truncated splice variants that promote dimerization are a common mechanism of resistance [109]. More recently, experimental evidence is emerging that BRAF V600 mutations can also signal as dimers. In cell lines transfected with a V600E mutant, dimeric V600E was isolated [110], whilst other studies have also demonstrated BRAF V600 mutants have a greater propensity to dimerise compared to wild type BRAF [111, 112]. Dimeric BRAF V600E may also more efficiently phosphorylate MEK compared to V600E models that cannot dimerise [113]. Class II mutations (e.g., K601E, G469A, BRAF fusions) in contrast function as constitutively active dimers, but still independently of RAS activation. When first-generation RAF inhibitors bind to one of the dimers, the induced structural changes reduce the affinity of drug binding to the second dimer, limiting clinical effectiveness [114]. Indeed, paradoxical activation can occur through transactivation of the non-inhibited protomer [115]. Next-generation BRAF inhibitors, such as tovorafenib, have been developed specifically to have activity against non-V600E mutations thought to signal as dimers [116]. The phase II FIREFLY-1 trial demonstrated the efficacy of tovorafenib in a cohort of paediatric low grade glioma patients – over 80% of whom harboured a BRAF fusion and would be resistant to first-generation RAF inhibitors – leading to accelerated Food and Drug Administration approval [117]. Class III mutations, on the other hand, possess impaired kinase activity; they activate MAPK signalling indirectly through enhanced RAS-dependent RAF heterodimerization, often with CRAF. As such, BRAF inhibitors cannot abrogate this CRAF mediated MAPK activation [108, 118].

The classification system of course does not capture the whole complexity of oncogenic BRAF mutations, and its limitations need to be considered. Understanding the molecular context in which a BRAF mutation occurs is also crucial in determining the best therapeutic response. In colorectal cancers with V600E mutations for example, first-generation BRAF inhibitors are ineffective in contrast to their efficacy in melanoma [106]. This is due to rapid feedback activation through epidermal growth factor receptor. As such, the addition of an epidermal growth factor receptor inhibitor to MAPK inhibitors is required [119].

Other strategies to overcome resistance have been or are being actively pursued. These include combining RAF inhibitors with upstream modulators such as src homology region 2 domain-containing phosphatase-2 or son of sevenless homolog 1 inhibitors, or downstream inhibitors targeting MEK and ERK. Dabrafenib, in combination with trametinib was approved after demonstrating clinical benefits compared to monotherapy in the COMBI-d and COMBI-v trials in advanced melanoma [120, 121]. Similarly, the combination of encorafenib and binimetinib gained approval due to positive outcomes from the COLUMBUS trial, with enhanced progression-free survival in metastatic melanoma compared to BRAF inhibitor monotherapy. Rational combination strategies involving pan-RAF inhibitors alongside inhibitors targeting parallel signalling pathways (e.g., PI3K/AKT) or cell cycle checkpoints are being investigated to mitigate compensatory resistance mechanisms and achieve more robust clinical outcomes.

The clinical utility of these inhibitors, while promising, is often tempered by significant toxicities, necessitating careful dose modification and management. Specifically, dermatological adverse events such as rash, pruritus, and photosensitivity are commonly observed with RAF inhibitors, while gastrointestinal toxicities, including diarrhoea and nausea, are prevalent with MEK inhibitors. Hepatotoxicity and cardiovascular complications, including cardiomyopathy, also have been reported, underscoring the need for a vigilant monitoring of hepatic function and cardiac function [122, 123]. Moreover, the combination of RAF and MEK inhibitors, though enhancing therapeutic efficacy, frequently exacerbates these toxicities (e.g grade 3 or higher events in 52% of patients with dabrafenib plus trametinib in the Combi-D trial) demanding meticulous management to maintain treatment adherence and optimise patient outcomes [120].

The advent of direct KRAS inhibitors, such as sotorasib and adagrasib, represents a recent breakthrough. Sotorasib, targeting the KRAS G12C mutation, gained approval following the CodeBreaK 100 and 200 trials, which showed significant clinical efficacy in non-small cell lung cancer patients previously treated with chemotherapy [124, 125]. Adagrasib, also targeting KRAS G12C, demonstrated promising results in tumour agnostic early-phase studies and has ongoing clinical trials investigating combinations with immune checkpoint inhibitors and other targeted therapies. The most common adverse events of KRAS inhibitors are diarrhoea, nausea and hepatotoxicity, but as a single agent treatment is generally well tolerated. This is highlighted by the low discontinuation rate (~10%) due to side effects in the Codebreak 200 trial [125].

Emerging pan-KRAS inhibitors aim to target broader KRAS mutation subsets, including KRAS G12D and KRAS G12V. Early-phase clinical trials are assessing their efficacy and safety profiles, potentially expanding the therapeutic landscape to include cancers currently resistant to specific KRAS inhibitors [126].

More recently, ERK inhibitors have entered clinical trials. Ulixertinib is currently in phase II trials, having shown signs of activity and acceptable pharmacokinetic profile in phase I evaluation [127]. However, the development of several other ERK inhibitors has been complicated by a lack of efficacy or safety concerns when used in combinations [128, 129].

## How can we successfully clinically target the MAPK pathway in advanced prostate cancer?

There have been limited attempts to target the RAF-MEK-ERK-RSK axis in advanced prostate cancer in the clinical setting. MEK inhibitors have been used with dramatic responses in case reports. One patient with a SND1-BRAF fusion had an excellent outcome to treatment with Trametinib [130]. In a second case report (unknown genomic background due to insufficient yield from pre-treatment biopsy) a patient that had progressed through all standardly available therapies at the time achieved a sustained 93% reduction in PSA and reversal of transfusion dependent anaemia. The patient sadly died of a stroke whilst remaining on Trametinib with no evidence of progression 18 months after initiation [29]. The National Cancer Institute-Match trial subprotocol R was a tumor-agnostic study arm that treated all-comers with non-V600 BRAF mutations with trametinib. One patient with a BRAF K601E mutated neuroendocrine prostate cancer achieved a near radiological partial response and over six months of disease control, an impressive result in an aggressive and difficult to treat subtype [131]. Whilst a larger phase II study of 14 patients receiving single agent Trametinib was terminated early for futility, patients were not selected for on the basis of MAPK pathway activation.

Overall, the evidence suggests that MAPK signalling is activated in a variety of contexts in patients with advanced metastatic prostate cancer, promoting growth and treatment resistance potentially in AR dependent and independent ways. Whilst a minority of prostate cancers will have activation through oncogenic mutations in MAPK proteins, the mechanisms of activation of the pathway are diverse, including autocrine and paracrine processes.

Given that BRAF mutations in prostate cancer are relatively uncommon (around 3%), and predominantly class II mutations when they do arise, it is unsurprising that first-generation BRAF inhibitors have not penetrated the treatment landscape. Mechanistic studies exploring the degree of pathway activation caused by these specific mutations in prostate cancer are lacking. Nevertheless, there may be utility of newer BRAF targeting agents in this subset of patients. Due to the rarity of BRAF mutations in prostate cancer, it is likely that clinical evaluation of these agents will rely on the read-out of tumour-agnostic studies.

Inhibiting at the level of MEK has shown some success in case studies, but lessons from other tumour types suggest that durable, long-term responses are unlikely to single agent MEK blockade [132]. It may be more advantageous to target even further downstream, at the level of the RSK family of proteins. Under normal circumstances, to become fully activated, RSKs require input from both phosphorylated ERK from the MAPK pathway, and 3-phosphoinositide-dependent kinase 1 from the PI3K pathway [133]. Hence, they sit at something of a convergence between two major signalling pathways, which may limit or delay resistance through MAPK/PI3K crosstalk commonly seen with MEK inhibitors [134]. Progress in targeting RSK clinically has been delayed due to the lack of specific inhibitors with acceptable pharmacokinetic and pharmacodynamic profiles, although newer compounds such as PMD-026, which is currently in phase I and II clinical trials, are overcoming these challenges [135]. Published data from the phase I trial shows an acceptable side effect profile, with minimal adverse events at the recommended phase II dose [136]. TAS0612 is a RSK, AKT and ribosomal protein S6 kinase inhibitor developed by Taiho, but a phase I study in solid tumours was terminated in part due to the side effect profile, highlighting the challenge of directly blocking two major signalling pathways concurrently [137].

In many cases, MAPK blockade is cytostatic rather than cytotoxic so enhancing the degree of apoptotic response could be an attractive combination strategy. This may also prevent the development of resistance through pathway cross-talk. In prostate cancers with dependency on the MAPK pathway, inhibiting the pathway is likely to affect both the expression and binding patterns of the BCL-2 family of proteins, creating therapeutic vulnerabilities that can be exploited to deliver cancer-cell specific kill. No studies to date have thoroughly evaluated the impact of MAPK pathway activation on the intrinsic apoptosis pathway in prostate cancer to inform potential clinical strategies.

Another potential approach could be to combine MAPK inhibition with AR pathway inhibitors, given the possibility that MAPK signalling promotes transcription of the AR programme and may promote AR pathway inhibitor resistance. There is already pre-clinical evidence demonstrating trametinib can reverse enzalutamide resistance, and that the RSK inhibitor PMD-026 enhances the activity of enzalutamide in 22Rv1 cells and xenograft models [30, 68].

However, for successful clinical translation of any of these potential strategies, it will be imperative to define and prospectively validate predictive biomarkers for MAPK inhibition in advanced prostate cancer. Additionally, given the resistance to single agent MAPK blockade usually seen, there is a need to identify the most promising synergistic combinations based on an understanding of the fundamental biology.

## Data Availability

All data is contained within the referenced publications.
